# Adolescent mental health education InSciEd Out: a case study of an alternative middle school population

**DOI:** 10.1186/s12967-018-1459-x

**Published:** 2018-04-03

**Authors:** Joanna Yang, Roberto Lopez Cervera, Susannah J. Tye, Stephen C. Ekker, Chris Pierret

**Affiliations:** 1 Clinical and Translational Science, Mayo Clinic, Rochester, MN USA; 2 Postbaccalaureate Research Education Program, Mayo Clinic, Rochester, MN USA; 3 Psychiatry and Psychology, Mayo Clinic, Rochester, MN USA; 4 Biochemistry and Molecular Biology, Mayo Clinic, Rochester, MN USA

**Keywords:** Mental health, (alternative) Education, Stigma, School-based, Adolescent

## Abstract

**Background:**

Mental illness contributes substantially to global disease burden, particularly when illness onset occurs during youth and help-seeking is delayed and/or limited. Yet, few mental health promotion interventions target youth, particularly those with or at high risk of developing mental illness (“at-risk” youth). Community-based translational research has the capacity to identify and intervene upon barriers to positive health outcomes. This is especially important for integrated care in at-risk youth populations.

**Methods:**

Here the Integrated Science Education Outreach (InSciEd Out) program delivered a novel school-based anti-stigma intervention in mental health to a cohort of seventh and eighth grade at-risk students. These students were assessed for changes in mental health knowledge, stigmatization, and help-seeking intentions via a classroom activity, surveys, and teacher interviews. Descriptive statistics and Cohen’s d effect sizes were employed to assess pre–post changes. Inferential statistical analyses were also conducted on pilot results to provide a benchmark to inform future studies.

**Results:**

Elimination of mental health misconceptions (substance weakness p = 0.00; recovery p = 0.05; prevention p = 0.05; violent p = 0.05) was accompanied by slight gains in mental health literacy (d = 0.18) and small to medium improvements in help-seeking intentions (anxiety d = 0.24; depression d = 0.48; substance d = 0.43; psychosis d = 0.53). Within this particular cohort of students, stigma was exceptionally low at baseline and remained largely unchanged. Teacher narratives revealed positive teacher views of programming, increased student openness to talk about mental illness, and higher peer and self-acceptance of mental health diagnoses and help-seeking.

**Conclusions:**

Curricular-based efforts focused on mental illness in an alternative school setting are feasible and integrated well into general curricula under the InSciEd Out framework. Preliminary data suggest the existence of unique help-seeking barriers in at-risk youth. Increased focus upon community-based programming has potential to bridge gaps in translation, bringing this critical population to clinical care in pursuit of improved mental health for all.

*Trial registration* ClinicalTrials.gov, ID:NCT02680899. Registered 12 February 2016, https://clinicaltrials.gov/ct2/show/NCT02680899

## Background

### The priority of mental health

Prevention and treatment of mental illness remain significantly underserved health needs, despite poor mental health being of worldwide concern [1]. Youth are uniquely vulnerable, as most mental illnesses have early onset [2]. Effective help-seeking remains low, and attitudinal barriers—particularly stigma and inadequate mental health literacy—are commonplace [3]. Regardless of established need, few stigma-reduction programs target adolescents [4], and broader promotion–prevention interventions are lacking [2]. Youth-centric programming is therefore a critical need given the formative and persistent role of adolescence in development.

### Theoretical frameworks to break down the barriers

While structural barriers to mental healthcare exist for broader policy intervention, knowledge and attitudes are readily addressable at the individual level. Improvements have been made in recent years to both study and bolster the public’s mental health literacy, but dissemination of accurate information remains a necessary focus for betterment of mental health [5, 6]. This is particularly true for youth, where mental health literacy efforts have traditionally been flagging [7]. One theoretical framework often applied to health interventions is the knowledge–attitude–behavior (KAB) model. In this model, increased knowledge can change attitudes and result in behavioral modification [8, 9]. The knowledge–attitude–behavior continuum plays a key role in modern definitions of mental health literacy with evidence to suggest that improved knowledge and reduced stigma can result in increased help-seeking and improved outcomes in mental health [10]. Meta-analysis additionally shows that educational programming can alter stigma toward mental illness, particularly for youth audiences [11].

Traditional health education at the bedside only works, however, if patients enter healthcare systems in the first place. Underserved populations who tend to not seek care may therefore be better reached though community-based methods [12, 13]. For youth, K-12 classrooms represent a unique community platform in health intervention due to the significant amount of time youth spend in school [14]. Yet, there is insufficient evidence for efficacy of school-based anti-stigma programming pertaining to mental illness [15]. Interventions targeting youth with mental illness or at high risk for developing mental illness are rare and comparatively unstudied. Lack of research evidence in this space is a priority, as it contributes to care fragmentation. Translational research is necessary to bridge the gap from bedside to curbside for the most vulnerable children and adolescents [16]. The study herein presents preliminary results from a school-based anti-stigma intervention in mental health piloted by one such translational program called Integrated Science Education Outreach (InSciEd Out).

### The InSciEd Out framework

InSciEd Out [17] is an intracurricular partnership that connects K-12 teachers with scientists and other health professionals to design school-based units targeting salient health topics. These units give real world context to selected state standards taught in the classroom and replace previous curricula targeting these standards. Under this novel framework, a foundation of science excellence for all students [18, 19] is extended to address student learning related to health, disseminating health education into the community. Because of this, InSciEd Out is a truly translational program that capitalizes upon longitudinal community engagement to help test, interpret, implement, and assess the impact of knowledge. These data transfer elements address a spectrum of translational science aimed toward behavioral changes and improved public health [20].

The InSciEd Out program is a community-based participatory research model where health topics of focus are chosen in collaboration with partnering schools and their community leaders. Selected teacher partners undergo a 12-day scientific and pedagogical internship before collaborating to design, implement, and assess their health-focused units. One key aspect of the produced lessons is that students are encouraged to be active creators rather than passive consumers of knowledge. This student-driven inquiry is at the heart of the health literacy that InSciEd Out fosters. Clinically-focused interventions using InSciEd Out’s health-related curricula are called prescription education [21] and are driven in part by the KAB health model. InSciEd Out’s mental health arm consequently seeks to improve attitudes and downstream behaviors toward mental illness through addressing mental health literacy.

Longitudinal study of InSciEd Out reveals sustained gains in science literacy, demonstrating capability of InSciEd Out-facilitated knowledge transfer [19]. An initial assessment of prescription education in influenza-focused curriculum found improvements in unprompted knowledge of preventative behaviors [21].

## Methods

### Study design

This pilot study presents findings from the inaugural run of InSciEd Out prescription education curriculum in mental health. Its quasi-experimental design follows a single cohort of seventh and eighth grade students at an InSciEd Out partner public alternative school in the Midwestern U.S. While alternative education is a broad term encompassing all nontraditional educational programming [22], the study school here voluntarily admits its ~ 350 students (grades 7–12) based on entrance criteria that include potential chemical dependence or mental health issues. These students are “at-risk” youth, defined as youth with diagnosed/diagnosable mental illness or who have a high likelihood of developing mental illness.

The intervention was a 20-day anti-stigma classroom experience in the 2015–2016 school year. Intervention lessons replaced non-mental health focused lessons addressing selected state standards in science, math, and language arts. The intervention unit culminated with mentored science research projects based on student-driven questions in mental health. One highlight of this curriculum was its integration of biogenetic explanations of mental illness with broader social and cultural context through a combination of education and contact programming (Table 1).

**Table 1:** Lesson components and target concepts

	Lesson	Target concepts	Strategies
Day 1	Introduction	Pre-evaluation and introductory activities	Engage Evaluate
Days 2–5	My brain and I	Biogenetic foundations (brain anatomy and neurotransmission) and psychosocial foundations (mental healthcare system and barriers) to explore topics of resiliency and nature vs. nurture	Explore Contact
Days 6–15	Mental health and I	Mental health research projects, experimental design and hypothesis generation, execution of scientific experiments, data analysis, creation of mental health promotion art	Explain Elaborate Extend
Days 16–18	Sharing my voice in mental health	Organization of mental health scientific research and artistic voice into multimedia presentation to share a personal story	Explain Elaborate Extend
Days 19–20	Conclusion	Post-evaluation and conclude activities	Explain Evaluate

The InSciEd Out mental health unit in this study first built a foundation of knowledge in mental health before facilitating student exploration of mental health topics with personal relevance

### Assessing student outcomes

This case study was hypothesis-generating by design due to its novel population. Nevertheless, it aimed to test the premise that students undergoing InSciEd Out mental health curriculum exhibit pre–post increases in mental health literacy, decreases in mental illness stigmatization, and increases in hypothetical mental health help-seeking behavioral intentions. Study metrics were chosen to align with the KAB theoretical framework. Classroom activities and student surveys were administered pre–post intervention. Pilot status, novelty, and lack of a comparable partner school drove the decision to not recruit an external control.

#### Misconceptions activity

A curriculum-embedded activity on mental health misconceptions used anonymous student polling to assess preliminary, cohort-level mental health literacy. Anonymous cohort polling precluded matched analyses. The activity incorporated ten misconceptions assembled from websites of repute [23–26] grouped into three categories, covering mental illness: (1) being fictitious, irrelevant, and caused by personal weakness (generally and specifically for substance use and depression); (2) having no recovery and no prevention; and (3) causing patients to be violent/unpredictable, be unintelligent, and need separation in healthcare.

#### Mental health surveys

Three previously published and validated adolescent surveys totaling 28 questions were selected to assess mental health knowledge, stigmatization, and help-seeking behavioral intentions. Reliability and validity testing were not completed in the study population due to restricted sample size and lack of a comparable population for validation.

There is no gold standard mental health knowledge inventory, particularly for youth. Here the study used the *Westbrook Mental Health Knowledge Test (WMHKT)*, a 13-question, true/false/not sure survey on knowledge of generalized mental health [27]. Analysis primarily concerned correctness of response without distinguishing between incorrect and “not sure” responses.

To assess whether knowledge changes were accompanied by attitudinal change, the *Adolescent Attribution Questionnaire (AQ*-*8*-*C)* measured stigmatizing attitudes [27, 28]. The AQ-8-C is the short-form, eight-question attribution questionnaire for assessment of public stigma. It measures stereotypes of blame, anger, pity, help, dangerousness, fear, avoidance, and segregation. Each question is rated on a 9-point Likert scale with question 7 (help) reverse coded. Higher scores indicate higher stigma, so pre–post change shows stigma reduction when negative. Primary analysis concerned overall stigma score with secondary analysis of each stereotype.

Lastly, the *General Help*-*Seeking Questionnaire Vignette Version (GHSQ*-*V)* was administered to assess whether knowledge and attitude changes extended to changes in intended behavioral modification. The GHSQ-V measures help-seeking intentions, health literacy, and perceived need for help [29]. Seven vignettes for different health conditions are posed, and respondents indicate their intention to seek help via 7-point Likert scales. Various help sources are queried for each scenario, and additional fields are provided for respondents to give a diagnosis (literacy) and to indicate whether they think the individual in the vignette needs help (perceived need). Preliminary analysis to generate a summative score for the GHSQ-V revealed highest scale reliability with exclusion of item I “would not see help” (Table 2). Missing responses were therefore filled in with the average value of responses in A–H (all other help sources) to facilitate total scoring. Help sources were not split into formal and informal sources due to small sample size and scale reliability. Analysis of fill-in responses for diagnoses accepted schizophrenia for psychosis, any reference to alcohol or drugs for substance misuse, and heart attack or stroke for heart disease.

**Table 2:** Creation of a summative score for the GHSQ-V

	Stress		Anxiety		Depression		Suicidal ideation		Substance misuse		Psychosis		Heart disease	
	Pre	Post	Pre	Post	Pre	Post	Pre	Post	Pre	Post	Pre	Post	Pre	Post
None	0.7471	0.6552	0.8473	0.6629	0.8523	0.8246	0.9189	0.7646	0.8645	0.6484	0.8550	0.6907	0.7627	0.6492
A	0.7277	0.6415	0.8459	0.6048	0.8311	0.7955	0.8985	*0.7998*	0.8551	*0.7109*	*0.8597*	*0.7055*	0.7622	0.5987
B	0.6967	0.6077	0.8189	0.6013	0.8314	0.8021	0.9045	0.7554	*0.8729*	0.6656	0.8505	0.6687	0.7178	0.5813
C	0.7417	0.5851	0.8457	0.6998	*0.8625*	0.8101	0.9187	0.7496	0.8691	0.5883	0.8427	0.6818	*0.7894*	0.7110
D	0.6785	0.5776	0.8197	0.5603	0.8195	0.8168	0.8949	0.7154	0.8270	0.6083	0.8375	0.6465	0.6990	0.5041
E	0.7456	0.5987	0.8162	0.5516	0.8357	0.7757	0.9041	0.6841	0.8172	0.5216	0.8174	0.6101	0.7102	0.4819
F	0.6904	0.5664	0.8318	0.6164	0.8356	0.7828	0.9126	0.7008	0.8303	0.6220	0.8355	0.6871	0.6929	0.5739
G	0.7153	0.6488	0.8302	0.6286	0.8353	0.7818	0.9096	0.7004	0.8283	0.5478	0.8328	0.5879	0.7597	0.6539
H	*0.7775*	0.6425	*0.8601*	0.6606	0.8376	0.8164	0.9097	0.7435	0.8700	0.6271	0.8489	0.6984	0.7576	0.6256
I	0.7208	*0.7361*	0.8046	*0.7367*	0.8390	*0.8638*	*0.9298*	0.7967	0.8629	0.6381	0.8281	0.6679	0.7445	*0.7436*

Cronbach’s alphas support exclusion of item “I” (reverse coded) to maximize scale reliability. Iterative reliability is calculated for the full scale and partial scales excluding each item. The option with highest reliability in each column is italicized

#### Teacher exit interviews

Semi-structured interviews were conducted post-curricular implementation with the two partner teachers to capture a larger narrative of student change. Questions were designed to explore teachers’ perceptions of (1) the curriculum, (2) metrics for assessing curricular effect, and (3) changes in student knowledge, attitudes, and behaviors. Interviews were recorded and transcribed before primary coding by two independent coders. Resultant coding was compared and condensed into a final coding manual for secondary coding into emerging themes.

### Statistical analyses

Of 19 eligible students, 14 assented to formal pre- and post-survey assessments. All participating teachers consented to interviews. Due to small sample size, primary results largely refer to changes in response distribution (average ± standard deviation, unless otherwise indicated). Effect sizes were calculated as Cohen’s d values from $d = \frac{{M_{post} - M_{pre} }}{{SD_{pooled} }}$, where $SD_{pooled} = \sqrt {\frac{{SD_{pre}^{2} + SD_{post}^{2} }}{2}}$. They are included for subscales for thoroughness but are subject to potentially low reliability and should be interpreted with caution. To supplement these descriptive statistics, inferential statistics were also conducted to provide a benchmark for future studies. Statistical tests were performed in JMP Pro 12 (2015). To be conservative, two-tailed p-values are reported. Misconceptions data was analyzed via Fisher’s exact test due to incompletely matched samples; average change in response on each survey inventory was assessed by Wilcoxon signed rank tests due to variable normality and small sample size.

## Results

### Mental health knowledge

The classroom activity, surveys, and teacher interviews painted a narrative of curricular effects. First, the misconceptions activity showed significant elimination of student misconceptions of mental illness (Table 3). At baseline, at least one student present indicated agreement with every misconception posed besides separation. By post-evaluation, none of the students present still agreed with any of the misconceptions, demonstrating with statistical significance (χ^2^, p = 0.00–0.05) for four of the statements posed that the InSciEd Out curriculum lowered students’ mental health misconceptions. After Bonferroni correction, misconception elimination for statement 5 was still statistically significant.

**Table 3:** Mental illness misconceptions

	Pre n = 18 (%)	Post n = 18 (%)	Fisher’s p-value
S1: Mental illnesses are not real illnesses	5.6	0.0	1.0000
S2: Mental illness will never affect me	5.6	0.0	1.0000
S3: People with mental illnesses are violent and unpredictable	27.8	0.0	*0.0455*
S4: Mental illness is caused by personal weakness	11.1	0.0	0.4857
S5: Addicts who cannot quit simply lack willpower	61.1	0.0	0.0001*
S6: A person who is depressed is just not trying hard enough to snap out of it	11.1	0.0	0.4857
S7: There is no recovery for people with mental health problems	27.8	0.0	*0.0455*
S8: It is impossible to lower the risk for developing mental illness	27.8	0.0	*0.0455*
S9: People with mental illness should be hospitalized away from other patients	0.0	0.0	1.000
S10: People with mental illness lack intelligence	22.2	0.0	0.1039

Students eliminate mental illness misconceptions post-InSciEd Out curriculum. Statements with significant change are italicized, and the statement that remains significant under multiple comparisons adjustment is asterisked

**Fig. 1:**
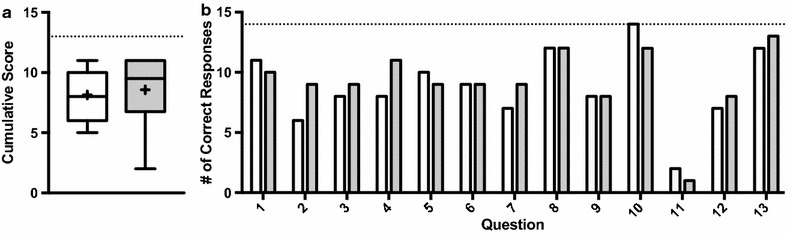
Westbrook Mental Health Knowledge Test. Students trend toward gains in mental health knowledge on the Westbrook Mental Health Knowledge Test post-intervention. Pre-scores are white; post-scores are grey; dotted lines indicate maximum possible score; “+” is the mean. **a** Cumulative score distribution. **b** Number of correct student responses, out of 14 eligible students, for each question

Analysis of the Westbrook Mental Health Knowledge Test partially corroborated this trend toward gains in mental health literacy. There was a shift toward higher post-scores in the negative skewness of the data (Fig. 1a). Individual item analysis of the number of students answering each question correctly (Fig. 1b) revealed that the largest gains were in questions 2 (“mental illness is like other diseases because a person who has it has symptoms that a doctor can diagnose”) and 4 (“the brain of a healthy person works the same as that of a mentally ill person”). Conversely, students scored poorest on question 11, which asked if “depression is a disease.” Matched change showed a 0.43 ± 2.59 increase in overall mental health knowledge from pre- (8.14 ± 2.07, median 8) to post- (8.57 ± 2.71, median 9.5) that was not statistically significant with an effect size of d = 0.18. GHSQ-V vignette identification was variable at baseline (stressed = 7%; psychosis = 21%; heart disease = 21%; anxiety = 29%; suicidal = 36%; depression = 43%; substance = 64%) and comparable at post-evaluation, excepting improvement in depression recognition (+ 36%).

### Mental illness stigmatization

**Fig. 2:**
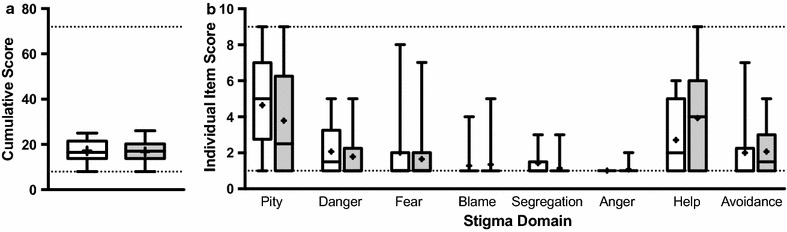
Adolescent Attribution Questionnaire. Student stigma is low at baseline and remains largely unchanged post-intervention. Pre-scores are white; post-scores are grey; dotted lines indicate minimum and maximum possible score; “+” is the mean. **a** Cumulative score distribution. **b** Score distribution for each of the different domains measured

The AQ-8-C extended results in knowledge to attitudinal shifts. Analysis revealed low baseline stigma that remained largely unchanged (Fig. 2a). This low baseline left nearly no room for stigma decrease post-intervention. Stigma was particularly low for the items of blame, segregation, and anger. The largest changes were seen in constructs of pity (− 0.86 ± 3.18) and help (1.21 ± 2.19) (Fig. 2b). Students scored 17.14 ± 4.80 pre- and 16.79 ± 4.79 post- for a matched stigma reduction of 0.36 ± 4.68 and an effect size of d = − 0.07 (Table 4). Effect sizes for individual stereotypes are provided in Table 4 for completeness but were heavily influenced by floor effects.

**Table 4:** Adolescent Attribution Questionnaire (AQ-8-C)

	Pre- Average (SD)	Post- Average (SD)	Difference Average (SD)	Wilcoxon p-value	d
Pity	4.64 ± 2.44	3.79 ± 2.94	− 0.86 ± 3.18	0.13	− 0.32
Dangerousness	2.07 ± 1.38	1.79 ± 1.19	− 0.29 ± 1.27	0.53	− 0.22
Fear	2.00 ± 2.04	1.64 ± 1.60	− 0.36 ± 2.06	1.00	− 0.20
Blame	1.29 ± 0.83	1.36 ± 1.08	0.07 ± 1.00	1.00	0.07
Segregation	1.43 ± 0.85	1.14 ± 0.53	− 0.29 ± 0.73	0.50	− 0.40
Anger	1.00 ± 0.00	1.07 ± 0.27	0.07 ± 0.27	1.00	0.38
Help	2.71 ± 1.98	3.93 ± 3.00	1.21 ± 2.19	0.06	0.48
Avoidance	2.00 ± 1.71	2.07 ± 1.33	0.07 ± 1.54	1.00	0.05
Total	17.14 ± 4.80	16.79 ± 4.79	− 0.36 ± 4.68	0.84	− 0.07

Student stigma is low at baseline and remains largely unchanged post-intervention

### Mental health help-seeking intentions

**Fig. 3:**
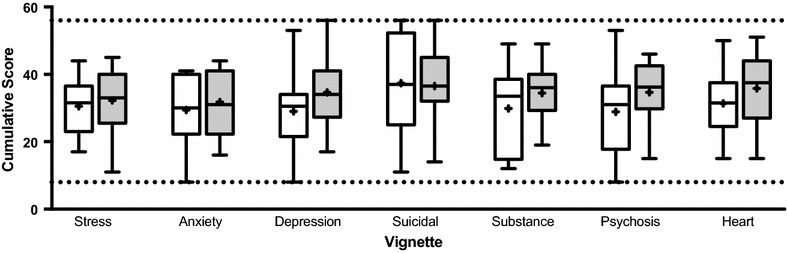
General Help-Seeking Questionnaire: Vignette Version. Students trend toward more centralized or increased help-seeking post-intervention. Pre-scores are white; post-scores are grey; dotted lines indicate minimum and maximum possible score; “+” is the mean

Ultimately, behavioral change is the outcome with highest clinical relevance. GHSQ-V data showed student responses trending toward increased hypothetical help-seeking intentions (Fig. 3). Responses became more centralized post-intervention with particular shortening of the interquartile range in the suicidal, substance, and psychosis vignettes. This regression to the mean is likely more representative of actual behavior and important for students who previously indicated a strong reluctance to seek help. Although none of the post-intervention changes were statistically significant under conservative two-tailed analyses, results in depression (Wilcoxon signed rank p = 0.12; d = 0.48) and psychosis (Wilcoxon signed rank p = 0.11; d = 0.53) approached statistical significance with additional small to medium effects in anxiety d = 0.24 and substance misuse d = 0.43 (Table 5). There appeared to be a general shift toward health help-seeking overall with a medium pre–post effect size for heart disease (d = 0.56). Perceived treatment need was high at baseline (79–100%) and remained largely unchanged post-intervention (86–100%).

**Table 5:** General Help-Seeking Questionnaire Vignette Version

	Pre- (Average ± SE)	Post- (Average ± SE)	Change (Average ± SE)	Wilcoxon p-value	d
Stress	30.55 ± 2.27	32.29 ± 2.53	1.74 ± 3.13	0.83	0.19
Anxiety	29.43 ± 2.75	31.79 ± 2.60	2.36 ± 3.67	0.79	0.24
Depression	29.00 ± 3.12	34.64 ± 3.19	5.64 ± 3.09	0.12	0.48
Suicidal	37.36 ± 4.12	36.50 ± 3.09	− 0.86 ± 2.92	0.28	− 0.06
Substance	29.86 ± 3.41	34.43 ± 2.20	4.57 ± 3.03	0.21	0.43
Psychosis	28.86 ± 3.31	34.67 ± 2.37	5.82 ± 3.02	0.11	0.53
Heart disease	31.36 ± 2.71	35.82 ± 2.90	4.46 ± 2.95	0.23	0.56

Students improve hypothetical help-seeking intentions post-InSciEd Out curriculum

### Teacher exit interviews

Overall, the teachers reported positive perceptions of the unit and of their students’ outcomes. The mental health content was relevant, and lessons with active inquiry were especially well received. In retrospect, surveys were not perceived to be the ideal source of data collection, particularly for a population of “reluctant learners” with “years and years of school failure.” One suggestion for future data collection was to conduct interviews with individual students to assemble a student-driven narrative. The teachers felt this might be a more sensitive metric of student growth.

Teachers reported numerous changes in student behaviors post-curricular implementation. Generally, the curriculum elicited high student participation. There were noted improvements to student willingness to risk their voice, both when talking about mental health and when presenting classwork to community members, teachers, and peers. Students were perceived to now have a common language to converse about mental health and consequently shared first/second-hand experiences more openly. Teacher 1 stated: “We would never hear [the students…] in the other years, or even at the beginning of the year […], own what they had,” but “because now they understand that these things all [fall] under mental illness, […] they [are] more willing to accept that that’s what they [have]. There [isn’t] that negative stigma attached to it.” Teacher 2 reported that students post-unit are “more tolerant of their classmates who have issues” while simultaneously encouraging peers to “do something” by seeking help. The teachers reported beliefs that their students now have a better idea of when to seek help for their mental health.

## Discussion

These preliminary findings show three trends meriting future study. First, at-risk adolescents may fundamentally differ from their peers. Baseline mental health literacy was significantly higher in this sample (8.14 ± 2.07) when compared to that of a previously published [27], general middle school population (6.87 ± 2.30) (GraphPad unpaired t-test p = 0.04; Table 6). Stigmatization was also lower in this at-risk cohort. One explanation could be prior exposure to the mental health system. Another rationale could be that there is a dimension of stigma unmeasured by the AQ-8-C. Establishing baseline literacy and stigma in this unique adolescent population is important, both to shed light on how to decrease care fragmentation and to tailor future health interventions to the needs of this vulnerable group.

**Table 6:** Reformatted Westbrook Mental Health Knowledge Test for Comparison

	Pre-test		Post-test	
	Correct	Not sure	Correct	Not sure
	n (%)	n (%)	n (%)	n (%)
Depression is the same thing as being sad. (F)	11 (78.6)	0 (0.0)	10 (71.4)	1 (7.1)
Mental illness is like other diseases because a person who has it has symptoms that a doctor can diagnose. (T)	6 (42.9)	4 (28.6)	9 (64.3)	2 (14.3)
Individuals who have a family member with a mental illness are more likely to have a mental illness themselves. (T)	8 (57.1)	3 (21.4)	9 (64.3)	2 (14.3)
The brain of a healthy person works the same as that of a mentally ill person. (F)	8 (57.1)	5 (35.7)	11 (78.6)	2 (14.3)
A person who does not get treatment for depression may feel better after a while, but there may be some long-lasting effects. (T)	10 (71.4)	2 (14.3)	9 (64.3)	4 (28.6)
How bad a person’s mental illness is depends on many things, including his or her genes and family environment. (T)	9 (64.3)	4 (28.6)	9 (64.3)	5 (35.7)
A person uses his or her brain to learn, but the heart controls a person’s feelings. (F)	7 (50.0)	5 (35.7)	9 (64.3)	5 (35.7)
Most people with mental illness can do normal things like go to school or work at a job. (T)	12 (85.7)	1 (7.1)	12 (85.7)	2 (14.3)
Treating mental illness can change the way the brain works. (T)	8 (57.1)	5 (35.7)	8 (57.1)	5 (35.7)
People with depression don’t need to see a doctor—they just get over it. (F)	14 (100.0)	0 (0.0)	12 (85.7)	1 (7.1)
Depression is a disease. (T)	2 (14.3)	2 (14.3)	1 (7.1)	2 (14.3)
There are no treatments that work for most mental illnesses. (F)	7 (50.0)	3 (21.4)	8 (57.1)	5 (35.7)
Students and other people who have a mental illness can’t learn. (F)	12 (85.7)	2 (14.3)	13 (92.9)	1 (7.1)
Total knowledge score	**Mean (SD)**	**Median**	**Mean (SD)**	**Median**
	8.14 ± 2.07	8.0	8.57 ± 2.71	9.5

Students improve their mental health knowledge on the Westbrook Mental Health Knowledge Test post-intervention. A detailed percentage breakdown is given here for ease of comparability to previous studies

Second, despite low inventory-reported stigma, teacher interviews suggest stigma remains a barrier for at-risk youth. More sensitive metrics, perhaps through individual interviews, are needed to better capture these students’ stories. This adolescent cohort may share more openly in venues prioritizing verbal communication, especially given positive reception to lessons involving open dialogue, project-based learning, and oral presentations. Clever data capture designed to be minimally invasive has the potential to draw out rich stories that cannot be easily gleaned from more traditional methods.

Lastly, this case study demonstrates potential feasibility and acceptability of curricular-based, anti-stigma mental health interventions for at-risk youth. This is an area of research that is sorely lacking in the current evidence base. Preliminary trends toward efficacy in mental health promotion make school-based partnerships a potentially efficient platform for health education dissemination. Mental well-being during childhood and adolescence is often inextricably tied to academic success. As such, schools have a vested interest in fostering open discourse about mental health amongst their students.

This pilot study is limited by its single cohort design and small sample size of a vulnerable population. To minimize risk, teacher exit interviews were conducted in lieu of student interviews, and actual student help-seeking behavior was not measured. An external control was not employed due to the preliminary nature of this study, the school-specific implementation of mental health curriculum, and lack of a comparable peer cohort. Random assignment was likewise not possible given the classroom environment, as InSciEd Out lessons are embedded into existent curricula. Future directions will work toward sample size enlargement facilitating recruitment of an external control, which will aid psychometric testing of existent tools and development of new assessment tools. In addition to this, upcoming studies will work with clinical partners to capture measures of actual student help-seeking behavior. Study metrics will also be monitored longitudinally with follow-up for retention.

## Conclusions

The pilot case study herein underscores the utility and potential of school-based mental health promotion efforts, particularly through InSciEd Out’s community-based framework. Targeted improvements in mental health literacy and moderate improvements in help-seeking intentions were accompanied by large decreases in mental health misconceptions. Inventory-reported stigma was low and largely unchanged. Nevertheless, teacher observations suggested anecdotal evidence of increased student self-identification with mental health diagnoses, openness to share, and peer acceptance.

Early and effective intervention is an undervalued tool for preventing or mitigating the negative, long-term consequences of poor health. Improved mental health for all can only be realized if affected individuals and their social support structures have sufficient literacy to recognize and understand mental illness, at least at a basic level. A necessary byproduct of this understanding is that stigma toward mental illness needs to decrease, or become eliminated, such that help-seeking is the norm rather than the exception. As such, anti-stigma interventions deserve heavy focus, particularly in high-risk populations. The students in this study represent a segment of general adolescents with demonstrated need for mental health promotion efforts. Although this study’s sample size is small, it is enriched for youth with increased risk of developing mental health issues. These students are difficult to reach in general K-12 populations and represent an underserved segment of adolescents.

Although care must always be exercised when working with vulnerable populations, community programming can no longer ignore at-risk youth simply because such studies are difficult to design and execute. Translational research pushing bedside to curbside has potential to give voice to the experiences and needs of this unique cohort in pursuit of improved mental health for all.

## Abbreviations

InSciEd Out: Integrated Science Education Outreach
WMHKT: Westbrook Mental Health Knowledge Test
AQ-8-C: Adolescent Attribution Questionnaire
GHSQ-V: General Help-Seeking Questionnaire Vignette Version
